# U. S. V. M. A., 1895

**Published:** 1895-10

**Authors:** 


### U.S.V.M.A., 1895.

The Convention of 1895 is now a matter of history, and passes into our records of progress with a wealth of pleasant memories. Aside from being the largest in numbers, the National organization has now cultivated a new territory of strength and power, that promises well to increase her usefulness and worth in every direction. It will bring to our Eastern meetings a stronger quota of the profession than ever before, and will in turn take to the West everyone who had the pleasure, intellectual entertainment, and fraternal privileges accorded and enjoyed at this gathering. It no longer need be doubted whether we can hold successful Western meetings, for Des Moines has answered this question for many years to come. The States contributary to the Mississippi sent more delegates this year than many of our Eastern meetings have contributed from the immediate territory where the conventions in the past held forth. Minnesota with an unusual delegation, Kansas, Missouri, Nebraska, Illinois, Indiana, and Ohio following, while Iowa, the home State, bringing to our sessions her votaries of the profession from every section of her State, made this meeting a memorable one. The interest, attention, earnestness, and thoroughness that characterized the reception of every report and paper was a rich contribution to our reputation as a useful organization. The good order, good feeling, and kindly spirit that prevailed under the most trying atmospheric conditions were as remarkable as it was a high honor to this body of veterinarians.

In the September issue of this JOURNAL we asked our readers and the members of the Association could they afford to stay away? To those who so decided we feel very regretful that they so done, for surely no one present at the various sessions but who has gone home to his clientage a broader and better-equipped veterinarian in every way. He has brushed up against his colleagues from North, South, East, and West, and he has heard a greater array of important every-day subjects considered in the broadest, fullest, and freest up-to-date light than has ever been considered before by any single convention of veterinarians in America.

